# High expression of ESRP1 regulated by circ-0005585 promotes cell colonization in ovarian cancer

**DOI:** 10.1186/s12935-020-01254-3

**Published:** 2020-05-19

**Authors:** Guanming Deng, Xiaofang Zhou, Le Chen, Ying Yao, Junjun Li, Yun Zhang, Chenhui Luo, Lijuan Sun, Jie Tang

**Affiliations:** 1 Department of Gynecologic Oncology, Hunan Cancer Hospital, The Affiliated Cancer Hospital Of Xiangya School of Medicine, Central South University, Yuelu District, 283 Tongzipo Road, Changsha, Hunan 410013 People’s Republic of China; 2 Department of Gynecology and Obstetrics, Union Hospital, Tongji Medical College, Huazhong University of Science and Technology, Wuhan, Hubei 430022 China; 3 Department of Gynecology and Obstetrics, The First People’s Hospital of Yueyang, Yueyang, Hunan 414000 China; 4 Department of Pathology, Hunan Cancer Hospital, The Affiliated Cancer Hospital of Xiangya School of Medicine, Central South University, Changsha, Hunan 410013 China; 5 Department of Animal Lab, Hunan Cancer Hospital, The Affiliated Cancer Hospital of Xiangya School of Medicine, Central South University, Changsha, Hunan 410013 China; 6 Department of Gynecology and Obstetrics, Shaoyang Central Hospital, Shaoyang, Hunan 422000 China; 7 Hunan Gynecologic Cancer Research Center, Hunan Cancer Hospital, The Affiliated Cancer Hospital of Xiangya School of Medicine, Central South University, Changsha, Hunan 410013 China

**Keywords:** ESRP1, Epithelial ovarian cancer, Metastasis, Colonization, MET

## Abstract

**Background:**

Ovarian cancer is the third most common gynecological cancer in the world but the leading cause of death among gynecological malignancies. Epithelial splicing regulatory protein-1 (ESRP1), a key negative splicing regulator in epithelial-mesenchymal transition (EMT), has been proven to be overexpressed and may plays a role in epithelial ovarian cancer (EOC) progression. However, the functional roles of ESRP1 and the underlying mechanisms in this process still remain unclear.

**Methods:**

Tumor invasion, migration, colony formation and animal experiments were used to study the malignant biological behavior of ESRP1. A vector-based system expressing circ-0005585 was established to investigate circRNA as a microRNAs sponge. RNA-Seq and cytoskeleton staining explored underlying mechanisms of ESRP1.

**Results:**

Our results demonstrated that circ-0005585 regulates ESRP1 overexpression via sponging miR-23a/b and miR-15a/15b/16. Overexpression of ESRP1 suppresses EOC cell migration, but promotes colonization and drives a switch from mesenchymal to epithelial phenotype (MET) in association with actin cytoskeleton reorganization, mainly by alternative splicing EPB41L5 and RAC1. Furthermore, we have shown that high ESRP1 expression may be associated with immune-suppression in tumor immune microenvironment in vivo.

**Conclusions:**

ESRP1 overexpression promotes MET status and correlates with actin cytoskeleton reorganization in EOC. ESRP1 plays an important role in EOC colonization. In addition, a miRs panel from two miR families can inhibit ESRP1, may provide an innovative approach for cancer theranostics.

## Background

Ovarian cancer is the leading cause of death among gynecologic malignancies. 90% of ovarian cancers are epithelial ovarian cancer (EOC), which carries a poor prognosis due to the advanced stage of disease at diagnosis, relatively unsuccessful treatment strategies, and a high rate of relapse [1]. Ovarian cancer cells are easily planted on peritoneum and abdominal organs, causing extensive metastases. The molecular mechanisms of ovarian cancer progression have not yet been elucidated, further hampering the diagnosis and treatment of ovarian cancer.

ESRP1, a key epithelial cell-specific RNA-binding protein, participates in EMT process by regulating alternative splicing of multiple genes, including CD44, CTNND1, ENAH, and FGFR2 [2, 3]. Previous studies have found that ESRP1 is highly expressed in ovarian cancer associated with a shorter patient survival [4]. It is closely related to cancer cell invasion and metastasis [5]. However, the factors leading to the high expression of ESRP1 in ovarian cancer are still unclear. And further studies are needed to elucidate the specific functions and mechanisms of ESRP1 on malignant biological behavior of ovarian cancer.

Highly conserved in evolution, microRNAs(miRs) are important post-transcriptional regulators of gene expression by direct base pairing to target sites within the 3’UTR region of messenger RNAs [6, 7]. The presence of miR sponge transcripts, referred to as competing endogenous RNA (ceRNA), has been shown to affect miRs activity. Several studies have shown that circRNA serves as a miRs sponge, controlling gene expression. For example, ciRS-7 contains more than 70 selectively-conserved miR target sites and strongly increases the level of miR-7 targets, making it an efficient miR-7 sponge in the human brain [8].

EMT-MET is a tightly regulated and complex dynamic process, which drive cancer cells to migrate from their primary tumor sites and re-colonized at distant sites [9, 10]. Pelvic dissemination is a predominant way for EOC cells to directly metastasize to adjacent organs. Down-regulating ESRP1 promoted the occurrence of EMT [4]. However, it remains unclear whether high expression of ESRP1 based post-transcriptional alternative splicing regulation of mRNAs could link ovarian cancer progression. In this study, we investigate the mechanisms that lead to up-regulation of ESRP1, as well as its downstream effects that lead to metastasis in EOC.

## Methods

### Patients and samples

Patients were included from Hunan Cancer Hospital/the Affiliated Cancer Hospital of Xiangya School of Medicine, Central South University, Changsha, China. Specimens included normal ovarian tissue, benign ovarian tumor tissue, primary and metastatic EOC tissue. 3 cases of normal ovarian tissue came from patients with non-ovarian cancer who have undergone ovariectomy. Benign tumor tissue were from 10 patients with benign ovarian tumors. 16 specimens of ovarian cancer were obtained from surgically resected tissue of ovarian cancer patients, and specimens of primary and metastatic lesions were collected. All study participants signed an informed consent form which was reviewed by the Institutional Review Board of Hunan Cancer Hospital. All EOC patients were treated with standard protocols in accordance with the NCCN (National Comprehensive Cancer Network) clinical practice guidelines for EOC by gynecological oncologists in the Hunan Cancer Hospital between 2017 and 2018. Surgical evaluation was used to determine the presence of metastases according to the 2014 International Federation of Gynecology and Obstetrics (FIGO) classification [11].

### Cell culture and transfection

The ovarian cancer cell lines SKOV3, A2780, HO8910, ID8 were purchased from the Type Culture Collection of the Chinese Academy of Sciences (Shanghai, China). All cells were cultured in Dulbecco’s modified Eagle’s medium (DMEM, HyClone, USA) supplemented with 10% fetal bovine serum (FBS, BI, Israel) and 1% penicillin–streptomycin solution (HyClone, USA) at 37 °C in a humidified atmosphere containing 5% CO2, as we described previously [12]. For lentivirus transfection (ESRP1 coding region sequence and vector map were shown in Additional file 1: Figure S1), SKOV3, A2780 or ID8 cells were incubated in a 24-well plate with 500 µl medium containing 20 µl (10^7^ U) lentivirus particles and 5 µg/ml polybrene for 24 h. Then changed the fresh medium once a day, and pumimycin (1 µg/µl) was added for 21 days to construct a stable cell line. Plasmid (circ-0005585 sequence and vector map were shown in Additional file 1: Figure S1) transfections were performed using Lipofectamine 3000 (Invitrogen, USA) according to the manufacturer’s instructions. miR mimics or scramble control RNAs (RiboBio, China, Catalog numbers were in Additional file 2: Table S1) were transfected into cells at a final concentration of 100 nM using a Lipofectamine 3000 according to the manufacturer’s instructions.

### RNA extraction, Reverse transcription and Quantitative real-time PCR

The mRNA level of each gene was measured via qRT-PCR. RNA was isolated using a total RNA Trizol Kit (Life Technologies, USA). cDNA synthesis was carried out using the RevertAid First Strand cDNA Synthesis Kit (Thermo Fisher Scientific, USA). mRNA was created using a mixture of oligodT and circular RNA adding random hexamers for priming. qRT-PCR for mRNA was conducted with ChamQ^TM^ SYBR Color qPCR Master Mix (Vazyme, China). The conditions were 95 °C for 30 s; 95 °C for 5 s, 60 °C for 30 s, and 39 cycles; the Melt Curve was 60.0 °C to 95.0 °C, increasing by 0.5 °C every 5 s. miRs were subjected to qRT-PCR using the Bulge-Loop^TM^ miRNA qRT-PCR Starter Kit(RiboBio, China). The conditions were 95 °C for 20 s; 95 °C for 10 s, 60 °C for 20 s, 70 °C for 5 s, and 40 cycles; the Melt Curve was 70 °C to 95 °C, with an increase of 0.4 °C per second. The data was analyzed using the ΔΔCt method and normalized to GAPDH levels [13]. The primer sequences used in qRT-PCR are presented in Additional file 2: Table S2.

### Western blot

Cells were washed with D-Hanks, after treatment and lysed in radio immunoprecipitation assay buffer (RIPA, Vazyme, China) which contained protease inhibitors (Roche, Germany). We quantified proteins by using the BCA Protein Assay Kit (Vazyme, China). Samples containing 20-30 μg of total protein were electrophoresed on SDS–polyacrylamide gels and transferred onto a PVDF membrane (Millipore, USA) by electroblotting using a BioRad Bis–Tris Gel system (Bio-Rad, USA). The membranes were blocked by 5% nonfat milk and incubated overnight with the primary antibody at 4 °C, followed by a brief wash with PBST, and subsequent incubation with a secondary antibody for 1 h at room temperature. Finally, ECL solution (Millipore, USA) was added to cover the blot surface. The signals were captured and the intensity of the bands was quantified by using the Bio-Rad ChemiDoc XRS + system (Bio-Rad, USA). Antibodies used for Western Blot included ESRP1 (ab107278, Abcam plc, Cambridge, MA, USA; concentration, 1: 300), GAPDH (23001, SAB, Baltimore, MD, USA; concentration, 1: 2000). Horseradish peroxidase-conjugated secondary antibodies goat anti-rabbit IgG were purchased from Servicebio (GB23303, Wuhan, Hubei, China; concentration, 1: 3000).

### Transcript stability assays

Cells were seeded at 2 × 10^6^ per well in 6-well plates. 24 h after seeding, Actinomycin D (BioRike, China) was added to the cells to a final concentration of 1 µg/ml. RNA was isolated at 0, 4, 8, and 12 h after the addition of Actinomycin D [14] using a total RNA isolation kit. Relative levels of the circRNA and line-RNA transcripts were assessed by qRT-PCR using different primers.

### Dual-luciferase reporter assay system

A luciferase reporter assay was performed in order to detect the direct binding of miR-15a/15b/16/23a/23b to ESRP1. The entire 3’-UTR of human ESRP1 (Sequences were in Additional file 1: Figure S1) was cloned into the pmirGLO vector (Additional file 1: Figure S1) and confirmed by sequencing to form a wild type luciferase reporter vector (ESRP1 WT 3’-UTR). To verify the binding specificity, the sequences of ESRP1 3’-UTR that bound with the miR-15a/15b/16/23a/23b were missed mutated (miR-15a/15b/16: GCTGCTA; miR-23a/23b: AATGTGAA), and it was also inserted into pmirGLO vector to generate a mutated luciferase reporter vector (ESRP1 mut 3’-UTR). For the luciferase reporter assays, HO8910 cells were plated in 96-well plates and then transiently co-transfected luciferase reporter vectors with miR-15a/15b/16/23a/23b-mimics or control mimics using Lipofectamine 3000. After transfection for 48 h, the relative luciferase activity was detected using a dual luciferase reporter assay system (Promega, USA). Luciferase activity was detected using an M200 microplate fluorescence reader (Tecan, Beijing, China), and the relative luciferase value was the firefly luciferase value normalized against Renilla luciferase activity.

### RNA-binding protein immunoprecipitation (RIP) assay

The immunoprecipitation of the circ-0005585 bound to Ago2 was performed using a Magna RIP™ RNA-Binding Protein Immunoprecipitation Kit (Millipore, USA). After transfection of different mimics for 48 h, 2 × 10^7^ HO8910 cells were harvested in RIP lysis buffer and the lysates were stored at − 80 °C. 8 µg of anti-Ago2 (ab32381, Abcam, UK) or normal control IgG (PP64B, RIP kit components, Millipore, USA) were incubated with magnetic beads for 2 h at RT and then 100 µl of the supernatant of RIP lysate was mixed with 900 µl of RIP immunoprecipitation buffer and added to the bead-antibody complexes to incubate overnight at 4 °C. The beads were further mixed with proteinase K buffer and incubated for 30 min at 55 °C and RNA was finally extracted for PCR use.

### Transwell migration and invasion assay

Transwell systems (Corning, USA) were used to evaluate the migration and invasion ability of cancer cells [12]. Briefly, for invasion assay, Matrigel (BD Biosciences, USA) was added to the upper surface of a polycarbonic membrane (pore size 8 μm) to form a thin gel layer to serve as the ECM. The upper compartment of the filter contained the treated cells at a density of 5 × 10^5^ cells/well in 200 μl of DMEM. The bottom filter was filled with 600 μl of conditional medium. After 24–48 h incubation at 37 °C with 5% CO2, the polycarbonic membrane was fixed with 4% paraformldehyde for 10 min and stained with 0.2% crystal violet solution. Then, the cells on the upper surface of the filter were removed by wiping with a cotton swab. Cells that had penetrated to the lower surface of the filter were counted under an Olympus microscope in three randomized fields at a magnification of 100 × . Cell migration assay was carried out in a transwell filter on membrane filters, which were not coated with matrigel.

### Colony formation assay

Cells (600 cells/well) were seeded into 6-well plates with DMEM medium supplemented with 10% FBS and cultured for 14 days. Then, colonies were fixed with methanol at room temperature for 15 min and stained with 0.2% crystal violet for 15 min, images were taken with a camera, and the total number of visible colonies were counted.

### Cell cytoskeleton staining

Normal or ESRP1 stably transfected SKOV3 and A2780 cells were washed with PBS and fixed with 4% paraformaldehyde for 10 min. Subsequently, cells were washed with PBS and incubated with 0.5% Triton X-100 for 5 min. After blocking with 1% bovine serum albumin, cells were incubated with TRITC-phalloidin (Yeasen, Shanghai, China) for 30 min. Then, their nuclei were dyed with DAPI for 5 min. Images were captured at 1000 × magnification by fluorescence microscopy.

### Transcriptome sequencing and analysis

1 × 10^7^ ESRP1-SKOV3 and EV(Empty Virus)-SKOV3 cells were cultured in DMEM medium and then RNA was extracted by Trizol. The quality of the RNA was checked by Agilent 2200 (Agilent, Santa Clara, CA, USA) and stored at − 80 °C. RNA with RIN > 8.0 indicated adequate quality. The RNA was then resuspended and wash with NEBNext Oligo dT magnetic beads, RNA Bingding Buffer, Wash Buffer and Tris in the NEBNext^®^ Poly(A) mRNA Magnetic Isolation Module kit (New England Biolabs, USA). RNA was inverted to cDNA using the NEB Next^®^ UltraTM Directional RNA Library Prep Kit for Illumina^®^ kit (New England Biolabs, USA) according to the instructions and purified using 1.8X AMPure XP magnetic beads (Beckman Coulter, USA). Adding a terminal repair reagent immediately after the PCR reaction, a linker was added, purified with AMPure XP magnetic beads, and subjected to fragment sorting using 0.8X, 0.2X AMPure XP magnetic beads. The final library was purified by amplification with AMPure XP magnetic beads after 15 cycles of amplification, then tested on an Agilent 2200 detector to meet the following requirements: 300 bp ≤ library main peak length ≤ 500 bp, concentration ≥ 2 ng/μl, peak type single, no miscellaneous peak. RNA sequencing was performed by Shanghai Novelbio Ltd. We applied the DESeq algorithm, using the criteria FC > 2 or < 0.5 and FDR < 0.05, to filter the differentially expressed genes. We use Fisher’s exact test for GO analysis based on the genes annotated in the comprehensive GO database. The KEGG pathway analysis of differential genes was performed by Fisher’s exact test, and FDR < 0.05 indicated significant differences. Gene co-expression network and alternative splicing bioinformatics analysis was assisted by Shanghai Novelbio Ltd. SKOV3 cells were injected into abdominal cavities of nude mice. Tumor tissues of three nude mice with intraperitoneal tumors were taken, and total RNA in the tissues was extracted with Trizol. RNA-Seq was then performed as described above. circRNA identification and quantitative analysis were performed separately to obtain circRNAs expressed in all three samples.

### Flow cytometry

The ascites obtained from C57 mice abdominal cavity, mononuclear lymphocyte were extracted by density gradient centrifugation, stained with anti-mouse CD8a-APC Antibody (BioLegend, USA), added and incubated for 30 min at room temperature in the dark. Finally, the cells were washed, immediately analyzed by flow Cytometry and analysis was done using FlowJo software.

### Mice

The female Balb/c nude mice and C57 mice, 5 to 7 weeks old, used in this experiment were purchased from Hunan SJA Laboratory Animal Co., Ltd (SJA, China). For nude mice, we injected 5 × 10^6^/200 μl/mouse ESRP1-SKOV3 or EV-SKOV3 cells into the peritoneal cavity with a syringe; for C57 mice, 1 × 10^7^/200 μl/mouse ESRP1-ID8 or EV-1D8 cells were injected into the peritoneal cavity; for subcutaneous tumor formation in C57 mice, 1 × 10^7^/200 μl/site ESRP1-ID8 or EV-ID8 cells were subcutaneously injected into the lower abdomen of each mouse. Mice were fed a regular diet and monitored closely. 4 weeks later, the mice were sacrificed, tumors were excised, ascites was collected and the volume and weight of each tumor were measured. The experimental protocol was approved by the Experimental Animal Ethics Review Committee of Hunan Cancer Hospital. To analyze infiltrating CD8 + T cells in tumors, we examined ESRP1, CD8 (ZCIBIO, China) and INHBE (Inhibin Subunit Beta E) in subcutaneous tumors of C57 mice by the immunohistochemical protocols previously described [4].

### Bioinformatics analysis

In this study, we downloaded dataset GSE18520 in the Gene Expression Omnibus (GEO) database. After regression calculation of the datasets by the affyPLM package in R language, weights, residuals and residuals sign images were drawn. The relative logarithmic expression, normalized unscaled standard errors and RNA degradation were detected. RMA algorithm was used to background correcting, normalizing and calculating expression of the datasets. We then converted the probe ID to gene symbol, k-Nearest Neighbor complemented the missing value. Limma package calculated the differentially expressed gene (logFC > 1 or logFC < −1, adj. P.Val < 0.05) [15]. Based on gene expression, correlations between genes were calculated using the cor.test() function in the psych package. In addition, we obtained the gene matrix of TCGA ovarian cancer samples from Firebrowse (http://firebrowse.org/) and the clinical information came from the published paper [16].

### Statistical analysis

All quantitative data were expressed as mean ± SEM values of three independent experiments unless otherwise indicated. Data from two groups were compared using the student’s T test. Correlation was analyzed using linear regression. Cumulative survival was calculated by the Kaplan–Meier analysis and the significance of the difference in survival was analyzed by Gehan-Breslow-Wilcoxon test. Results were considered statistically significant if p < 0.05. All data were statistically analyzed using graphpad prism (GraphPad Software Inc., La Jolla, CA).

## Results

### Up-regulating ESRP1 induced MET and promoted colonization

Our recent paper found that ESRP1 was highly expressed in ovarian cancer compared to normal or benign ovarian tissue [4]. The high ESRP1 expression group had higher advanced FIGO stage, higher tumor grade and higher proportion of residual tumor ≥ 1 cm than the low ESRP1 expression group. Survival rates in the low ESRP1 expression group were significantly longer than in the high ESRP1 expression group [5].

**Fig. 1:**
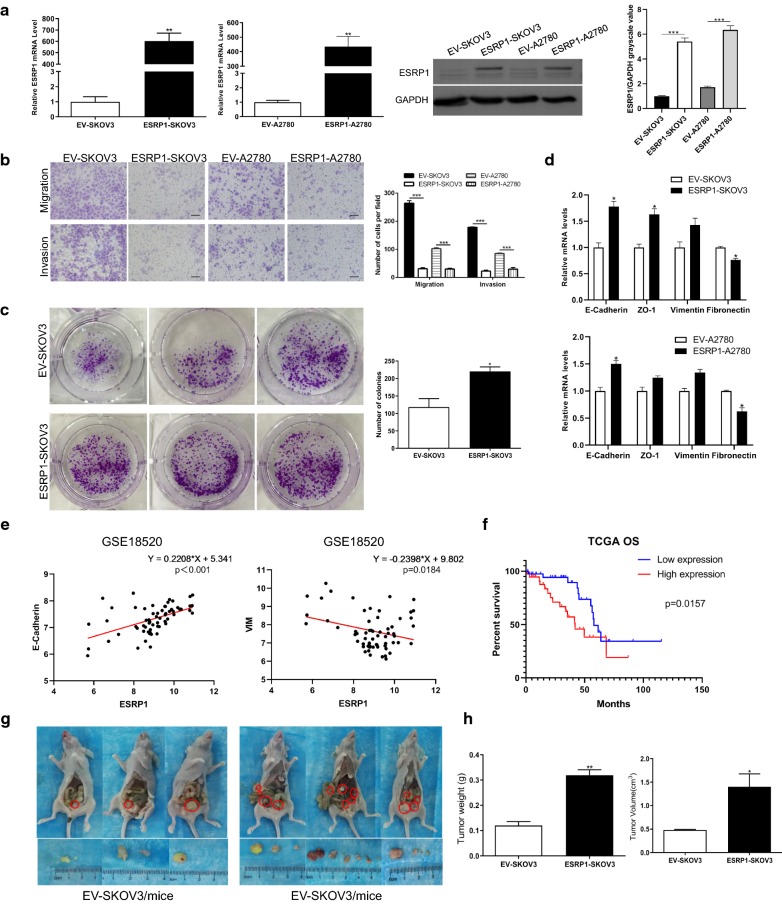
ESRP1 induced MET, inhibited invasion and migration, promoted colonization of EOC cells. **a** ESRP1 expression in ESRP1-SKOV3 and ESRP1-A2780 cells was confirmed by qRT-PCR and western blot. GAPDH served as a control. **b** ESRP1-SKOV3, ESRP1-A2780 and the control groups (EV-SKOV3, EV-A2780) migration and invasion assay using a Corning Transwell System. Scale bar 100 μm. Histogram represented cell count. **c** ESRP1-SKOV3 and EV-SKOV3 cells clone forming assay. Histogram showed the count of cell colonies after cultured for 14 days. **d** Epithelial markers E-Cadherin, ZO-1 and mesenchymal markers Vimentin, Fibronectin were detected in ESRP1-SKOV3 and ESRP1-A2780 compared to the control groups (EV-SKOV3, EV-A2780) by qRT-PCR. **e** ESRP1 was positively correlated with E-Cadherin and negatively correlated with VIM in dataset GSE18520. **f** Kaplan–Meier curve showed overall survival of patients with ovarian cancer without residual disease in stage III and IV in TCGA database. High level ESRP1 group (red, n = 39) or low level ESRP1 group (blue, n = 39). **g** ESRP1/EV-SKOV3 cells were injected into the abdominal cavity of nude mice to construct a orthotopic xenograft model of ovarian cancer. Red circles marked macroscopic tumor tissue. **h** Histograms showed the tumor weight and volume in nude mice of experimental and control groups. *P<0.05, **P<0.01, ***P<0.001. Error bars indicate SEM

We constructed ESRP1 overexpression cell lines ESRP1-SKOV3 and ESRP1-A2780 using lentivirus. Compared with the control groups EV-SKOV3 and EV-A2780, ESRP1 mRNA and protein in ESRP1-SKOV3 and ESRP1-A2780 cells were significantly increased (Fig. 1a). Transwell chamber was used to detect cell migration and invasion. We found that ESRP1-SKOV3 and ESRP1-A2780 cells migrated from the bottom membrane of Transwell chamber significantly less than the control groups (p < 0.001). After adding Matrigel to the basement membrane surface of the chamber, ESRP1-SKOV3 and ESRP1-A2780 cells passing through the basement membrane were still significantly less than the control groups (p ≤ 0.001) (Fig. 1b). This indicated that ESRP1 can inhibit migration and invasion of SKOV3 and A2780 cells. Colony-forming ability of ESRP1-SKOV3 and EV-SKOV3 cells was detected by plate cloning assay. ESRP1-SKOV3 cells formed significantly more colonies than EV-SKOV3 after the same number of cells were cultured for 14 days (p < 0.05) (Fig. 1c). We used CCK-8 assay to detect the cell proliferation of ESRP1-SKOV3, ESRP1-A2780 cells and the control groups, but there was no significant difference between the two groups (Additional file 3: Figure S2d). ESRP1 alternatively spliced and regulated epithelial cells. Compared with EV-SKOV3, in ESRP1-SKOV3 epithelial marker—E-Cadherin, ZO-1—was significantly increased (p < 0.05), and the mesenchymal marker Fibronectin was significantly decreased (p < 0.05). Similarly, E-Cadherin was significantly increased (p < 0.05) and Fibronectin was significantly decreased (p < 0.05) in ESRP1-A2780 cells compared with EV-A2780 cells (Fig. 1d). In addition, ESRP1 was significantly positively correlated with epithelial marker E-Cadherin (p < 0.01), and negatively correlated with the mesenchymal marker VIM (p < 0.05) (Fig. 1e) in GSE18520 dataset.

In order to clarify the effect of ESRP1 on prognosis of ovarian cancer patients, we screened TCGA ovarian cancer samples [16], stage III and IV, absence of macroscopic disease after tumor cytoreductive surgery. Patients with high level of ESRP1 were found to have significantly shorter overall survival (OS) compared to those with low expression (Fig. 1f).

ESRP1 also promoted EOC cells progression in vivo. ESRP1-SKOV3 and EV-SKOV3 cells were injected into the peritoneal cavity of nude mice to construct a orthotopic xenograft model of ovarian cancer. After 4 weeks, nude mice were sacrificed. Tumor size, number of nodules, and presence of metastatic lesions in ESRP1-SKOV3 group were significantly higher than the control group (Fig. 1g). Weight and volume of tumor nodules dissected from the ESRP1-SKOV3 group were also significantly greater than those of the control group (p < 0.05) (Fig. 1h). The results indicated that ESRP1 had a significant role in promoting the development of ovarian cancer in vivo. circ-0005585 adsorbed 2 miR families that target ESRP1.

**Fig. 2:**
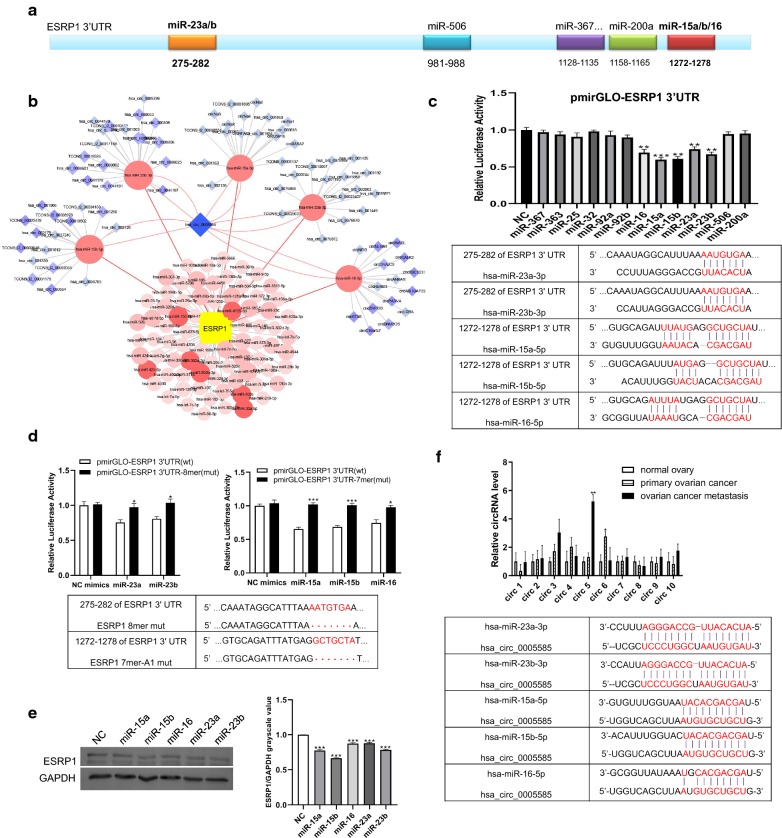
circ-0005585 bound to miRs targeting ESRP1. **a** miR binding sites in ESRP1 3’ UTR. **b** circRNAs (blue diamonds) compete with miRs (red circles) to regulate ESRP1. **c** Luciferase reporter assay was used to screen individual miR that may regulate ESRP1 and the binding sites of ESRP1 3’UTR to miRs: miR-23a/b and miR-15a/15b/16. **d** Dual luciferase reporter assay validated the inhibition of miR-23a/b and miR-15a/b/16 on wild-type(wt) and mutant(mut) ESRP1 3’UTR. Red dots represented base deletion mutations. **e** Western blot demonstrated the inhibitory effect of miRs on ESRP1. Histogram represented quantification of bands. **f** qRT-PCR verified 10 circRNAs in normal ovarian, primary ovarian cancer and metastatic carcinoma tissue. And the binding sites of circ-0005585 to miRs. The official name of circ5 in circBase was obtained by sequence matching. circ5 RNA-Seq subject ID was chr5_43704400_43675511_ + 28889-NNT. CircBase ID was hsa_circ_0005585. *P<0.05, **P<0.01, ***P<0.001. Error bars indicate SEM

Why ESRP1 was highly expressed in ovarian cancer remained unclear. To determine the up-regulation mechanism, we investigated the non-coding RNA regulation mode upstream of ESRP1. TargetScan [17] and miRanda [18] were used to predict miRs targeting ESRP1. Based on the score and site type, we screened 13 miRs (Additional file 3: Figure S2a) targeting ESRP1 3’ UTR, and identified different binding sites for miR families (Fig. 2a). Figure 2b showed the overall screening process. The abundance of these 13 miRs was obtained by ovarian cancer miRs profile analysis in TCGA database. Except miR-367, other miRs were relatively abundant (Additional file 2: Table S3). ESRP1 3’UTR sequences were recombined into the dual luciferase reporter vector pmirGLO. Each miR mimics and recombinant plasmid pmirGLO-ESRP1 3’ UTR were co-transfected into HO8910 cells. Luciferase activity was significantly reduced after transfection of miR-23a, miR-23b, miR-15a, miR-15b, miR-16 (Fig. 2c). These five miRs have complementary binding sites to ESRP1 3’ UTR. miR-23a and miR-23b are in the same family; miR-15a, miR-15b and miR-16 are in another family (Fig. 2c). To verify the inhibitory effect of miRs on ESRP1, we mutated the miRs binding sites of ESRP1 3’UTR, then constructed recombinant dual luciferase reporter vectors. Recombinant vectors and miRs were co-transfected into HO8910 cells. Compared with the wild type(wt) vector, luciferase activity in the mutant(mut) recombinant vector was increased (Fig. 2d). When these five miRs were transfected into HO8910 cells alone, ESRP1 protein was detected significantly decreased by Western Blot (Fig. 2e).

**Fig. 3:**
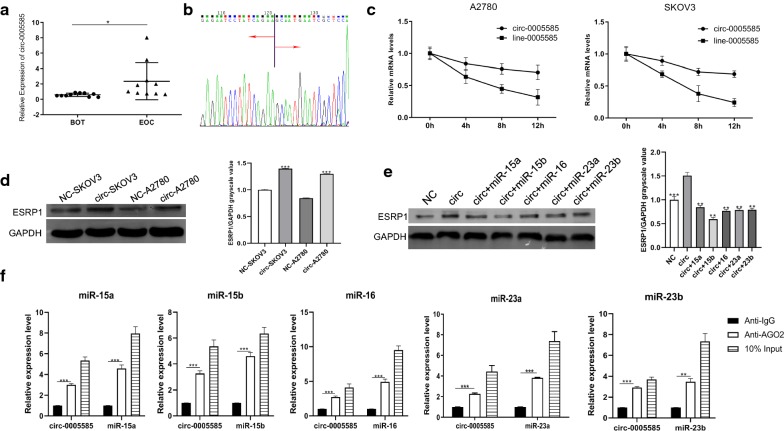
circ-0005585 competitively bound miRs to up-regulate ESRP1. **a** Clinical samples validation of circ-0005585 expression in BOT (Benign Ovarian Tissue, n = 10) and EOC (Epithelial Ovarian Cancer, n = 10). **b** circ-0005585 DNA sequencing, red arrow represented circulation direction. **c** Actinomycin D was added in A2780 and SKOV3 cells to inhibit transcription. Then the stability of circ-0005585 was verified by comparing with line-0005585. d Western blot showed that circ-0005585 promoted ESRP1. Histogram represented quantification of bands. **e** circ-0005585 was transfected into HO8910 cells alone or co-transfected with miRs to examine its effect on ESRP1 by Western blot. Histogram represented quantification of bands. **f** RNA immunoprecipitation determined the sponge absorption of circ-0005585 on miR-15a/15b/16 and miR-23a/23b. *P<0.05, **P<0.01, ***P<0.001. Error bars indicate SEM

Due to its conservation and stability, circRNA has been extensively studied in cancer in recent years [19]. We injected SKOV3 cells into the peritoneal cavity of nude mice to mimic the formation and degradation of non-coding RNA during tumor cell growth. Tumor tissues with good growth were obtained from nude mice. High-throughput sequencing was performed to obtain circRNAs stably expressed in all samples. We screened 10 circRNAs (Additional file 3: Figure S2b) that served as sponges for miR-23a/b and miR-15a/15b/16. Verification was performed in normal ovary, primary ovarian cancer and ovarian cancer metastasis specimens. circ5 was highly expressed in metastastic tumors (p < 0.01), and circ6 was highly expressed in primary tumors (p < 0.05). We chose circ5 for research object because ESRP1 was highly expressed in ovarian cancer tissue, and ovarian cancer had characteristics of extensive abdominal cavity metastasis. The official name of circ5 is obtained by sequence matching. Circ5 RNA-Seq subject ID is chr5_43704400_43675511_ + 28889-NNT. CircBase ID is hsa_circ_0005585 (Fig. 2f).

circ-0005585 up-regulated ESRP1 by competitively binding miRs. circ-0005585 was expressed higher in EOC specimens (n = 10) than BOT (n = 10, Fig. 3a). Primers were designed according to the loop-forming characteristics of circRNA, and DNA sequencing was performed after PCR amplification, which confirmed the loop-forming property of circ-0005585 (Fig. 3b). Adding actinomycin D to inhibit transcription, we found that circ-0005585 was more stable than line-0005585 (Fig. 3c).

After transfection of circ-0005585, ESRP1 protein was significantly increased in SKOV3 and A2780 cells (p < 0.001) (Fig. 3d). Co-transfection of circ-0005585 with miRs in HO8910 cells revealed that co-transfection did not affect the transcription of circ-0005585 (Additional file 3: Figure S2c). circ-0005585 was co-transfected with miR-23a/b and miR-15a/15b/16, which counteracted the promotion effect of circ-0005585 on ESRP1 (Fig. 3e). RNA immunoprecipitation further confirmed that circ-0005585 competitively bound miRs (Fig. 3f) and indirectly promoted ESRP1.

### High expression of ESRP1 induced MET through alternative splicing in EOC

**Fig. 4:**
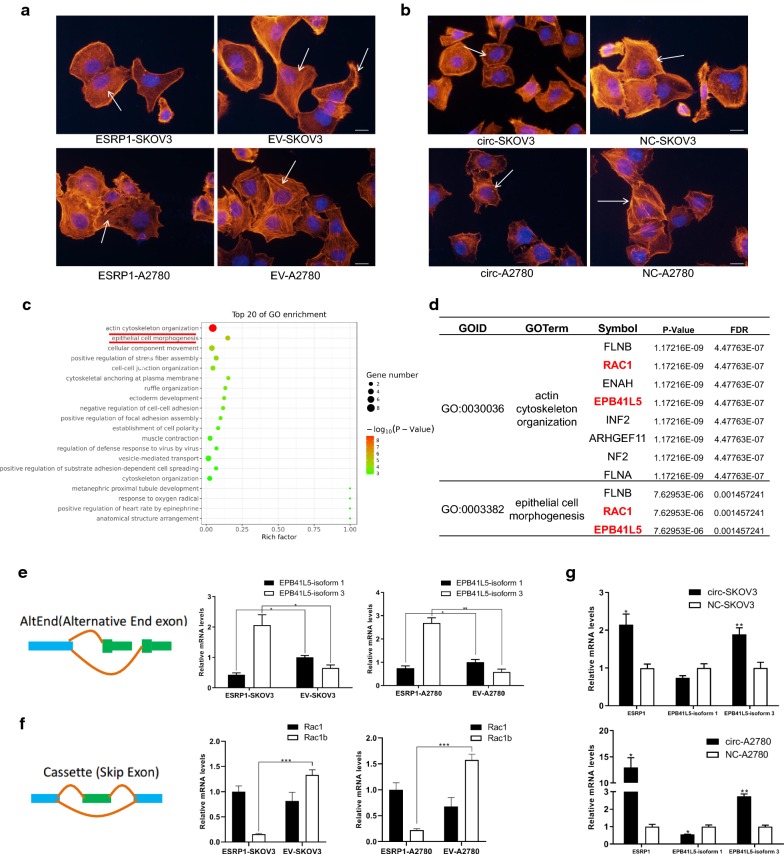
ESRP1 alternative spliced cytoskeleton to promote MET process. **a, b** Phalloidin staining was used to dye F-actin to display morphological changes in ESRP1 overexpression EOC cells (**a**) and circ-0005585 transfected SKOV3 and A2780 cells (**b**). Phalloidin staining (red) indicated F-actin. DAPI staining (blue) indicated nucleuses. Scale bar 10 μm. **c** GO enrichment terms of differential genes in ESRP1-SKOV3 cells compared with EV-SKOV3. **d** ESRP1 alternative splicing differential genes were mainly enriched in two GO terms: actin cytoskeleton organization and epithelial cell morphogenesis. **e** ESRP1 regulated the subtypes of the cytoskeleton-associated protein EPB41L5 by alternative splicing the end exon, validated in ESRP1-SKOV3 and ESRP1-A2780 by qRT-PCR. **f** ESRP1 regulated cytoskeleton-associated protein Rac1 by exon skipping and verified by qRT-PCR in the SKOV3 and A2780 cells. **g** Transfection of circ-0005585 up-regulated ESRP1 and promoted alternative splicing EPB41L5 in ESRP1-SKOV3 and ESRP1-A2780. *P<0.05, **P<0.01, ***P<0.001. Error bars indicate SEM

To observe changes in cytoskeleton during mesenchymal-epithelial transition (MET), we stained the cytoskeleton F-actin with phalloidin. ESRP1-SKOV3 and ESRP1-A2780 cells’ cytoskeletal arrangement became more turbulent compared with the control groups (Fig. 4a), similarly existed in cells transfected with circ-0005585 (Fig. 4b).

In order to study the specific mechanism of ESRP1 in inhibiting cell migration and invasion, promoting cell colony formation and cytoskeleton changes, we extracted RNA from ESRP1-SKOV3 and EV-SKOV3 cells, and then performed high-throughput sequencing. 38 differential genes with alternative splicing events were screened by False Discovery Rate (FDR) < 0.05. Gene Ontology (GO) analysis of these differential genes revealed that the two most different GO biological processes were actin cytoskeleton organization and epithelial cell morphogenesis (Fig. 4c). The differential genes contained in these two terms were shown in Fig. 4d. The complete results of GO analysis can be found in Additional file 3: Figure S2e. Kyoto Encyclopedia of Genes and Genomes (KEGG) pathway analysis were shown in Additional file 3: Figure S2f. ESRP1 alternative splicing regulated EPB41L5 by Alternative End exon (AltEnd) mode (Fig. 4e). ESRP1-SKOV3 and ESRP1-A2780 cells had significantly lower EPB41L5-isoform 1 (p < 0.05), and higher EPB41L5-isoform 3, than EV-SKOV3 and EV-A2780 cells (p < 0.05). Both RAC1 and FLNB were involved in alternative splicing by exon skipping (or cassette exon). We chose the more classical RAC1 to study (Fig. 4f) and found that Rac1b was significantly reduced in ESRP1-SKOV3 and ESRP1-A2780 cells (p < 0.05). In SKOV3 and A2780 cells, EPB41L5-isoform 3 was also significantly increased after transfection with circ-0005585 (p < 0.05) (Fig. 4g).

### ESRP1 was associated with immune-suppression in tumor microenvironment in vivo

**Fig. 5:**
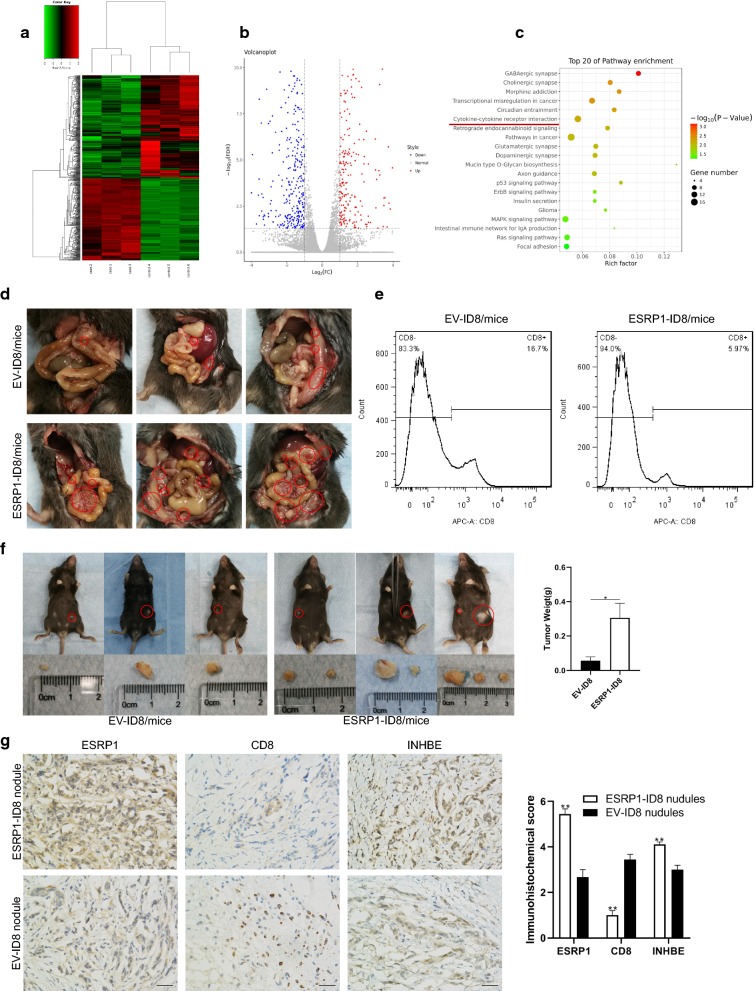
ESRP1 was associated with immunesuppression in tumor microenvironment. **a** Heatmap showed the RNA-Seq differentially genes in ESRP1-SKOV3 and EV-SKOV3. **b** Volcanoplot indicated the distribution of differential genes by RNA-Seq. Blue dots indicated downregulation (log_2_FC < −-1, FDR < 0.05) in ESRP1-SKOV3. Red dots indicated upregulation (log_2_FC > 1, FDR < 0.05). **c** KEGG pathway analysis of differential genes screened by RNA-Seq. **d** ESRP1/EV-ID8 cells were injected intraperitoneally into C57 mice. Red circles marked macroscopic tumor tissue. **e** Ascites from two groups of C57 mice were collected, and the amount of CD8 + T cells was detected by flow cytometry. **f** ESRP1/EV-ID8 cells were injected subcutaneously into C57 mice. Red circles marked tumor tissue, and the histogram showed tumor weight. **g** Immunohistochemistry was used to detect the expression of ESRP1, CD8 and INHBE in subcutaneous nodules from C57 mice. Scale bar 50 μm. *P<0.05, **P<0.01, ***P<0.001. Error bars indicate SEM

High ESRP1 promoted MET and enhanced cell colony-forming of EOC cells. To study the main regulatory mechanisms between them, we extracted RNA from ESRP1-SKOV3 and EV-SKOV3 cells for RNA sequencing. Based on the amount of gene transcriptome expression between two groups, we screened 624 differential genes with log2FC < −1 or log2FC > 1 (FC, Fold Change) and FDR < 0.05. The heat map (Fig. 5a) and volcano map (Fig. 5b) showed the distribution of differential genes as a whole. We analyzed the enrichment pathways of differential genes and found that there were significant differences in the cytokine–cytokine receptor interaction pathway (p < 0.05) (Fig. 5c). Comprehensive analysis of all differential pathways was conducted to study the correlation between pathways (Additional file 4: Figure S3a), which revealed that the major regulatory pathways downstream of ESRP1 included cytokine–cytokine receptor interaction, focal adhesion and wnt signaling. Cytokine-cytokine receptor interaction was at the core. The image (Additional file 4: Figure S3b) showed differential genes in the cytokine–cytokine receptor interaction pathway. Compared with the control group, TRAIL, GITRL, APRIL of the TNF family and INHBE of the TGF-β family were significantly increased in ESRP1-SKOV3 cells (p < 0.05). Therefore, it was suggested that high ESRP1 may be associated with tumor immune suppression.

To demonstrate the immunosuppressive effect of ESRP1 in tumor immune microenvironment in vivo. We injected ESRP1-ID8 cells into the peritoneal cavity of C57 mice. It was found that ESRP1-ID8 was widely metastasized in the peritoneal cavity, and many small tumor nodules were formed on the mesenteric, omental, peritoneal and abdominal organs (Fig. 5d). However, only a small number of tumor nodules were found on the peritoneal and mesenteric surface in EV-ID8 group (Fig. 5d). The ascites of two groups were collected, and mononuclear cells in ascites were extracted. Changes of CD8 + cells were detected by flow cytometry. The results showed that CD8 + cells in ascites of the ESRP1-ID8 group were significantly reduced compared to the control group (Fig. 5e). Because tumor nodules in C57 mouse abdominal cavity were small and adhered to surrounding connective tissue too tightly to separate, we injected ESRP1-ID8 and EV-ID8 cells subcutaneously in C57 mice to observe the changes of infiltrating CD8 + T cells and INHBE in tumor nodules. Results showed that the weight of subcutaneous tumor nodules in ESRP1-ID8 group was significantly heavier than the EV-ID8 group (Fig. 5f). Immunohistochemistry (IHC) was performed on tumor tissues. ESRP1 and INHBE in ESRP1-ID8 group were significantly higher than in the control group (p < 0.05), but CD8 was significantly lower (p < 0.05) (Fig. 5g). These results indicated that ESRP1 may increased the secretion of INHBE, thereby reducing the infiltration of CD8 + T cells in tumor microenvironment and promoting tumor growth in vivo.

## Discussion

In this study, which combined bioinformatics, biochemical and functional approaches as well as clinical data, we characterized a novel post-transcriptional network that links cancer colonization and immune-suppression in tumor microenvironment regulated by high ESRP1 expression in EOC.

ESRP1 is an epithelial-specific splicing factor discovered recently [2, 3]. It mainly regulates genes related to intercellular adhesion, actin cytoskeleton, cell polarity and cell migration at the post-transcriptional level by alternative splicing [20]. In stem cells, the ESRP1-CD44v-xCT axis contributes to the attenuation of oxidative stress [21]. It also plays an important role in the development and progression of cancers [5, 22–24]. Previous studies have shown that in patients with ER (Estrogen Receptor) positive breast cancer, OS was significantly shorter in the high ESRP1 group [23]. In melanoma, the lower the expression of ESRP1, the longer the patient’s survival. And the lower expression of ESRP1 was also associated with increased immunocytotoxicity, indicating that ESRP1 can be used as a prognostic marker [25]. Furthermore, alternative splicing of CD44 mRNA by ESRP1 enhances metastasis in lung cancer [22]. However, the cancer-promoting effect of ESRP1 has not been conclusive, especially in ovarian cancer.

Most of the previous studies focused on down-regulating ESRP1 to promote EMT in cancers [26–28]. In EMT, ESRP1 was thought to directly regulate these subtypes such as FGFR2, ENAH, CD44 and CTNND, thereby losing cell-to-cell adhesion and polarity, increasing cancer cell invasion and migration [2–4]. While ESRP1 was significantly up-regulated in EOC, there was a lack of relevant cancer cell biology behaviorial studies. We found that overexpression of ESRP1 inhibited migration and invasion and promoted colony formation of ovarian cancer cells. K Horiguchi et al. [29] found that NMuMG cells overexpressing ESRP1 changed from spindle to cobblestone-like shape and E-Cadherin was up-regulated. We also observed that the cytoskeleton arrangement was disordered and the pseudopods were reduced in ESRP1 overexpressed EOC cells. These cells were transformed from fusiform to elliptical. E-Cadherin was up-regulated, indicating that MET occurred. We obtained the alternative splicing network downstream of ESRP1, mainly including: actin cytoskeleton organization and epithelial cell morphogenesis. Two major factors, Rac1 and EPB41L5, were at the core. Rac1b is an alternative splicing isoform of Rac1 and contains functional region exon 3b. Hiroki Ishii et al. [20] found that ESRP1 knockdown up-regulated Rac1b expression leading to filamentous pseudopod formation and enhanced cell motility in head and neck squamous cell carcinoma (HNSCC) cells. We found that Rac1b was significantly down-regulated in ovarian cancer cells overexpressing ESRP1. EPB41L5 is involved in the cancer mesenchymal program [30]. It encodes 2 isoforms: the long subtype has a region that binds to paxillin (PBS) [31], while the short subtype loses this region. Up-regulating ESRP1 in the mesenchymal breast cancer cell line, MDA-MB-231, resulted in a significantly higher expression of the short subtype of EPB41L5 [2]. In our study, EPB41L5-isoform 1 (long subtype) was decreased after up-regulating ESRP1 in EOC cells, while EPB41L5-isoform 3 (short subtype) was significantly upregulated. We speculate that ESRP1 alternatively splicing the cytoskeleton-associated proteins Rac1 and EPB41L5, resulting in changes in the cytoskeleton and accompanying changes in cell morphology, resulting in a cell transition from a mesenchymal phenotype to an epithelial phenotype.

Furthermore, we shed light on the mechanism leading to high expression of ESRP1 in ovarian cancer. Previous studies were able to show correlation between ESRP2 expression and DNA methylation or gene copy number in EOC, but the same was not done for ESRP1 [5]. Through extensive bioinformatics analyses, we validated five miRs, miR-23a/b and miR-15a/15b/16 with an inhibitory binding site to the ESRP1 3’UTR, which target ESRP1 and belong to two families. Through high-throughput sequencing, we found a large number of circRNAs present in ovarian cancer. Many studies have shown that circRNA was rich, stable and highly conserved in eukaryotic cells and was widely involved in gene regulation [32]. It also plays an important role in tumor development [33]. circ-0005585 was found based on competitive binding site with miR23a/b and miR-15a/15b/16. It was transcribed from NNT exons and was 623 bp in length. As a highly prevalent circRNA, circ-0005585 sponging miRs has been proven a stable mechanism for up-regulating ESRP1 in EOC.

Immune microenvironment is an important part of the tumor microenvironment. It is mainly composed of tumor-infiltrating lymphocytes (TILs) and other immune cells [34, 35]. Intratumoral infiltration of T lymphocytes was positively correlated with the prognosis of ovarian cancer [36]. Studies [25] have classified TCGA melanoma cases into ESRP1-low,–truncated and–full-length groups based on ESRP1 expression. The ESRP1 low expression group expressed mesenchymal markers, and had high immunocytolysis activity, more PD-L2 and CTLA-4 expression, also had a better survival rate. We found that the cytokine–cytokine receptor interaction pathway was changed in high ESRP1 group. More interestingly, INHBE was detected significantly increased while CD8 was decreased in mice tumor tissues. INHBE, which belongs to the transforming growth factor-β (TGF-β) family, might be substantially involved in the pathogenesis and malignant transformation in human endometrium [37]. Tumor microenvironment plays a vital role in ovarian cancer metastasis [38], which partly determines the malignant biological behavior of cancer cells, but also provides potential therapeutic targets [39–41]. ESRP1 promoted colonization during EOC cell metastasis. Thus, we hypothesized that high-ESRP1 EOC cells may inhibit the infiltrating CD3 + CD8 + T cells by secreting the immunosuppressive cytokine INHBE in tumor microenvironment, thereby inhibiting tumor immunity and promoting tumor cell colonization and growth.

## Conclusions

In order to find the molecular mechanism that leading to high expression of ESRP1 in ovarian cancer cells, we determined that circ-0005585 increased ESRP1 by competitively binding to miR-23a/b and miR-15a/15b/16. To clarify the carcinogenic effect of ESRP1 in ovarian cancer, we found that ESRP1 inhibited cancer cell migration and invasion by alternatively splicing cytoskeleton-associated proteins Rac1 and EPB41L5, and promoted colonization and colony formation. In addition, ESRP1 can promote the secretion of INHBE. In summary, ESRP1 plays a comprehensive role in progression of ovarian cancer. Our study provides a new understanding of the underlying biology of ovarian cancer by which high ESRP1 could lead to aggressive colonization in EOC. Our data deepens the understanding of the mechanism of EOC metastasis, may offer candidate targets for precise therapy in patients with ovarian cancer.

## Supplementary information

**Additional file 1: Figure S1.** Plasmid vectors and cDNA sequences.

**Additional file 2: Tables S1–S3.** Catalog numbers of miRs mimics, primer sequences and ESRP1-related miRs matrix.

**Additional file 3: Figure S2.** Non-coding RNA list and ESRP1 bioinformatics functional analysis.

**Additional file 4: Figure S3**. ESRP1-related pathways.

## Data Availability

The datasets supporting the conclusions of this article are included within the article (and its additional files). RNA-Seq data are deposited publically in the NCBI Gene Expression Omnibus (GEO) database, including raw circRNAs sequencing data (GSE133676, https://www.ncbi.nlm.nih.gov/geo/query/acc.cgi?acc=GSE133676) and ESRP1/EV-SKOV3 cells transcriptome data (GSE133841, https://www.ncbi.nlm.nih.gov/geo/query/acc.cgi?acc=GSE133841). Additional dataset used were previously published and the GEO accession numbers was GSE18520 [42].

## Abbreviations

EOC: Epithelial ovarian cancer
EMT: Epithelial-mesenchymal Transition
MET: Mesenchymal-epithelial transition
ESRP1: Epithelial splicing regulatory protein 1
IHC: Immunohistochemistry
FDR: False discovery rate
qRT-PCR: Quantitative real-time PCR
CCK-8: Cell counting Kit-8
OS: overall-survival
ER: Estrogen receptor
GO: Gene ontology
KEGG: Kyoto Encyclopedia of Genes and Genomes
AltEnd: Alternative end exon
TCGA: The cancer genome atlas
GEO: Gene expression omnibus
