# Supplementary material for: High expression of ESRP1 regulated by circ-0005585 promotes cell colonization in ovarian cancer
Source: Cancer Cell Int. 2020 May 19;20:174. doi: 10.1186/s12935-020-01254-3 (PMC7236301; doi:10.1186/s12935-020-01254-3)
Supplement: Supplementary file 1 — Additional file 1: Figure S1. Plasmid vectors and cDNA sequences. [file 12935_2020_1254_MOESM1_ESM.pdf]

a

CTCGAGGGGCCCCAGCAGTTAGAACATCCTCAGAAAAGAAGTGTTTGAAAGATGTATGGTGATCTTGAAACCTCCAGACACAAGAAAACTTCTAGCAAATTC
AGGGGAAGTTTGTCTACACTCAGGCTGCAGTATTTTCAGCAAACTTGATTGGACAAACGGGCCTGTGCCTTATCTTTTGGTGGAGTGAAAAAATTTGAGCTA
GTGAAGCCAAATCGTAACTTACAGCAAGCAGCATGCAGCATACCTGGCTCTTTGCTGATTGCAAATAGGCATTTAAAATGTGAATTTGGAATCAGATGTCTCCA
TTACTTCCAGTTAAAGTGGCATCATAGGTGTTTCCTAAGTTTTAAGTCTTGGATAAAAACTCCACCAGTGTCTACCATCTCCACCATGAACTCTGTTAAGGAAG
CTTCATTTTTGTATATTCCCGCTCTTTTCTCTTCATTTCCCTGTCTTCTGCATAATCATGCCTTCTTGCTAAGTAATTCAAGCATAAGATCTTGGAATAATAAAATC
ACAATCTTAGGAGAAAGAATAAAATTGTTATTTTCCCAGTCTCTTGGCCATGATGATATCTTATGATTAAAAACAAATTAAATTTTAAAACACCTGAAGATAAATTA
GAAGAAATTGTGCACCCTCCACAAAACATACAAAGTTTAAAAGTTTGGATCTTTTTCTCAGCAGGTATCAGTTGTAAATAATGAATTAGGGGCCAAAATGCAAA
ACGAAAAATGAAGCAGCTACATGTAGTTAGTAATTTCTAGTTTGAACTGTAATTGAATATTGTGGCTTCATATGTATTATTTTATATTGTACTTTTTTCATTATTGAT
GGTTTGGACTTTAATAAGAGAAATTCCATAGTTTTTAATATCCCAGAAGTGAGACAATTTGAACAGTGTATTCTAGAAAACAATACACTAACTGAACAGAAGTGA
ATGCTTATATATATTATGATAGCCTTAAACCTTTTTCCTCTAATGCCTTAACTGTCAAATAATTATAACCTTTTAAAGCATAGGACTATAGTCAGCATGCTAGACTGA
GAGGTAAACACTGATGCAATTAGAACAGGTACTGATGCTGTCAGTGTTTAACACTATGTTTAGCTGTGTTTATGCTATAAAAGTGCAATATTAGACACTAGCTAG
TACTGCTGCCTCATGTAACTCCAAAGAAAACAGGATTTCATTAAGTGCATTGAATGTGGATATTTCTCTAAGTTACTCATATTGTCCTTTGCTTGAATGCAATGC
CGTGCAGATTTATGAGGCTGCTATTTTTATTTTCTGTGCATTACTTTAACACCTTAAAGGGAGAAGCAAACATTTCCTTCTTCAGCTGACTGGCAATGGCCCTTT
AACTGCAATAGGAAGAAAAAAAAAAAGGTTTGTGTGAAAATTGGTGATAACTGGCACTTAAGATCGAAAAGAAATTTCTGTATACTTGATGCCTTAAGATGCCC
AAAGCTGCCCAAAGCTCTGAAAGACTTTAAGATAGGCAGTAATGCTTACTACAATACTACTGAGTTTTTGTAGAGTTAACATTTGATAATAAAACTTGCCTGTTT
AATCTCAAGTCGAC

b

ATGACGGCCTCTCCGGATTACTTGGTGGTGCTTTTTGGGATCACTGCTGGGGCCACCGGGGCCAAGCTAGGCTCGGATGAGAAGGAGTTGATCCTGCTGT
TCTGGAAAGTCGTGGATCTGGCCAACAAGAAGGTGGGACAGTTGCACGAAGTGCTAGTTAGACCGGATCAGTTGGAACTGACGGAGGACTGCAAAGAAG
AAACTAAAATAGACGTCGAAAGCCTGTCCTCGGCGTCGCAGCTGGACCAAGCCCTCCGACAGTTTAACCAGTCAGTGAGCAATGAACTGAATATTGGAGTA
GGGACTTCCTTCTGTCTCTGTACTGATGGGCAGCTTCATGTCAGGCAAATCCTGCATCCTGAGGCTTCCAAGAAGAATGTACTATTACCTGAATGCTTCTATT
CCTTTTTTGATCTTCGAAAAGAATTCAAGAAATGTTGCCCTGGTTCACCTGATATTGACAAACTGGACGTTGCCACCAATGACAGAGTATTTAAATTTTGAGAA
GAGTAGTTCAGTCTCTCGATATGGAGCCTCTCAAGTTGAAGATATGGGGAATATAATTTTAGCAATGATTTCAGAGCCTTATAATCACAGGTTTTCAGATCCAG
AGAGAGTGAATTACAAGTTTGAAAGTGGAACTTGCAGCAAGATGGAACTTATTGATGATAACACCGTAGTCAGGGCACGAGGTTTACCATGGCAGTCTTCAG
ATCAAGATATTGCAAGATTCTTCAAAGGACTCAATATTGCCAAGGGAGGTGCAGCACTTTGTCTGAATGCTCAGGGTCGAAGGAACGGAGAAGCTCTGGTT
AGGTTTGTAAGTGAGGAGCACCGAGACCTAGCACTACAGAGGCACAAACATCACATGGGGACCCGGTATATTGAGGTTTACAAAGCAACAGGTGAAGATTT
CCTTAAAATTGCTGGTGGTACTTCCAATGAGGTAGCCCAGTTTCTCTCCAAGGAAAATCAAGTCATTGTTCGCATGCGGGGGCTCCCTTTCACGGCCACAG
CTGAAGAAGTGGTGGCCTTCTTTGGACAGCATTGCCCTATTACTGGGGGAAAGGAAGGCATCCTCTTTGTCACCTACCCAGATGGTAGGCCAACAGGGGA
CGCTTTTGTCCTCTTTGCCTGTGAGGAATATGCACAGAATGCGTTGAGGAAGCATAAAGACTTGTTGGGTAAAAGATACATTGAACTCTTCAGGAGCACAGC
AGCTGAAGTTCAGCAGGTGCTGAATCGATTCTCCTCGGCCCCTCTCATTCCACTTCCAACCCCTCCCATTATTCCAGTACTACCTCAGCAATTTGTGCCCCC
TACAAATGTTAGAGACTGTATACGCCTTCGAGGTCTTCCCTATGCAGCCACAATTGAGGACATCCTGGATTTCCTGGGGGAGTTCGCCACAGATATTCGTAC
TCATGGGGTTCACATGGTTTTGAATCACCAGGGCCGCCCATCAGGAGATGCCTTTATCCAGATGAAGTCTGCGGACAGAGCATTTATGGCTGCACAGAAGT
GTCATAAAAAAAACATGAAGGACAGATATGTTGAAGTCTTTCAGTGTTCAGCTGAGGAGATGAACTTTGTGTTAATGGGGGGCACTTTAAATCGAAATGGCTT
ATCCCCACCGCCATGTAAGTTACCATGCCTGTCTCCTCCCTCCCTACACATTTCCAGCTCCTGCTGCAGTTATTCCTACAGAAGCTGCCATTTACCAGCCCTC
TGTGATTTTGAATCCACGAGCACTGCAGCCCTCCACAGCGTACTACCCAGCAGGCACTCAGCTCTTCATGAACTACACAGCGTACTATCCCAGCCCCCCAG
GTTCGCCTAATAGTCTTGGCTACTTCCCTACAGCTGCTAATCTTAGCGGTGTCCCTCCACAGCCTGGCACGGTGGTCAGAATGCAGGGCCTGGCCTACAAT
ACTGGAGTTAAGGAAATTCTTAACTTCTTCCAAGGTTACCAGTATGCAACCGAGGATGGACTTATACACACAAATGACCAGGCCAGGACTCTACCCAAAGAA
TGGGTTTGTATTTAA

c

GCAATGAATCGCTCCCTGGCTAATGTGATTCTTGGAGGCTATGGCACCACTTCAACAGCTGGTGGAAAACCCATGGAAATTTCTGGCACACATACGGAAATC
AACCTTGACAATGCAATTGACATGATTCGAGAAGCTAATAGCATTATTATTACACCAGGCTATGGTCTCTGTGCAGCCAAAGCTCAATACCCCATTGCTGATTT
GGTAAAGATGCTCACTGAGCAAGGCAAAAAAGTCAGGTTTGGAATTCACCCAGTTGCAGGCCGAATGCCTGGTCAGCTTAATGTGCTGCTGGCTGAGGCT
GGTGTGCCATATGACATTGTGTTGGAAATGGATGAGATCAACCATGATTTTCCAGATACTGATTTGGTCCTTGTAATTGGAGCTAATGACACTGTTAATTCAGC
AGCTCAAGAAGATCCCAACTCTATTATTGCAGGCATGCCAGTCCTTGAGGTCTGGAAATCAAAGCAGGTGATTGTTATGAAGAGGTCTTTGGGTGTTGGCTA
TGCTGCAGTGGACAATCCAATCTTCTACAAACCTAACACGGCCATGCTTCTAGGTGATGCCAAGAAAACATGTGACGCGCTCCAGGCGAAAGTTAGAGAAT
CCTATCAGAA

d

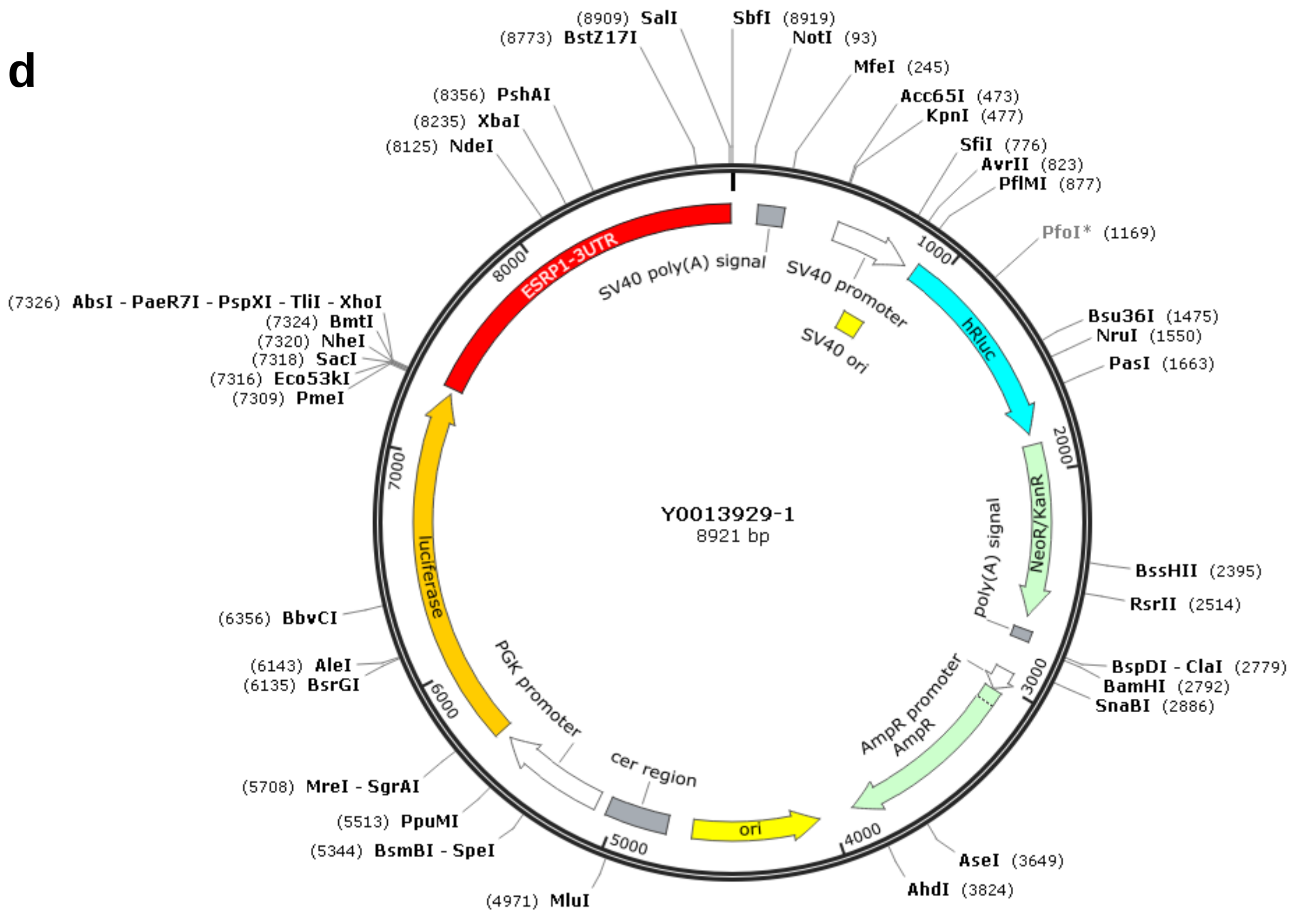

e

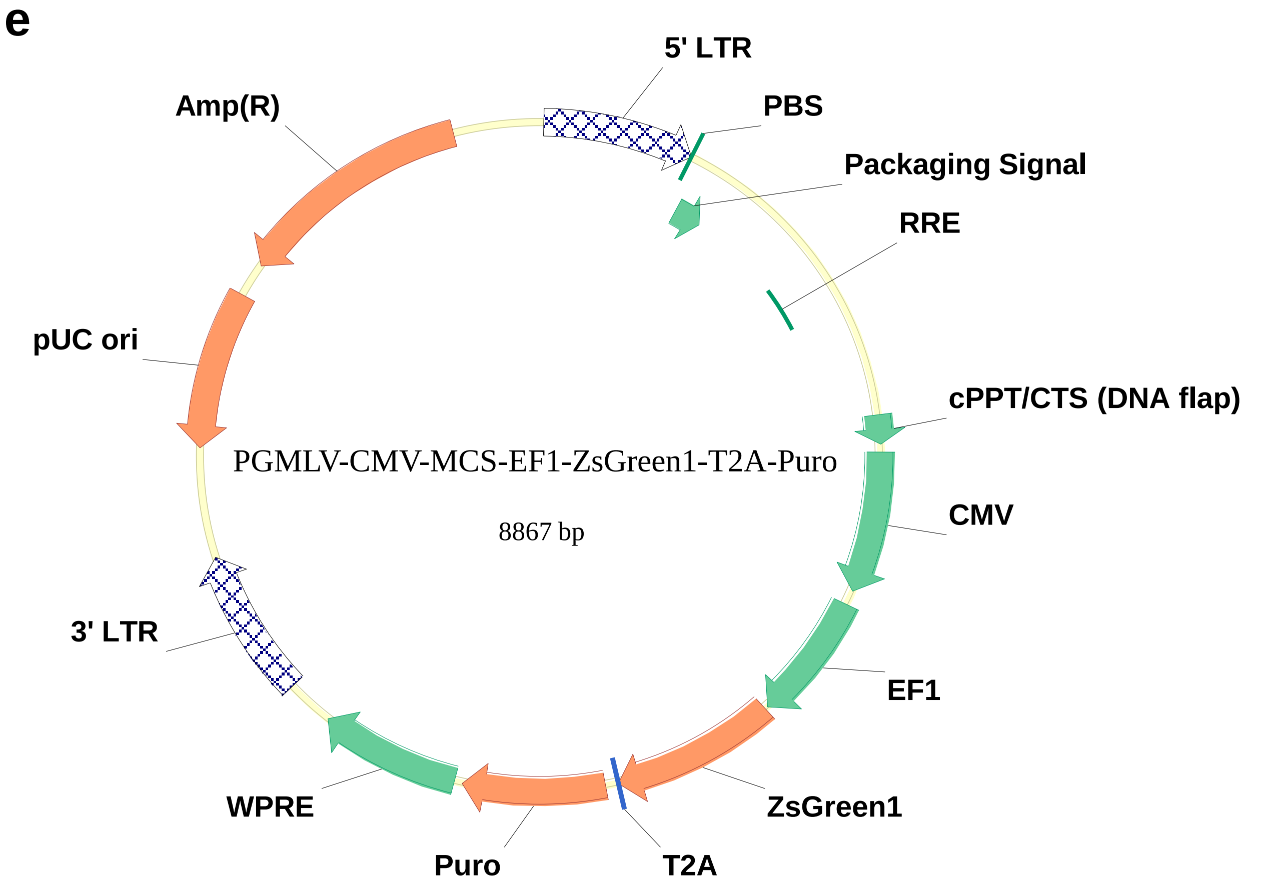

f

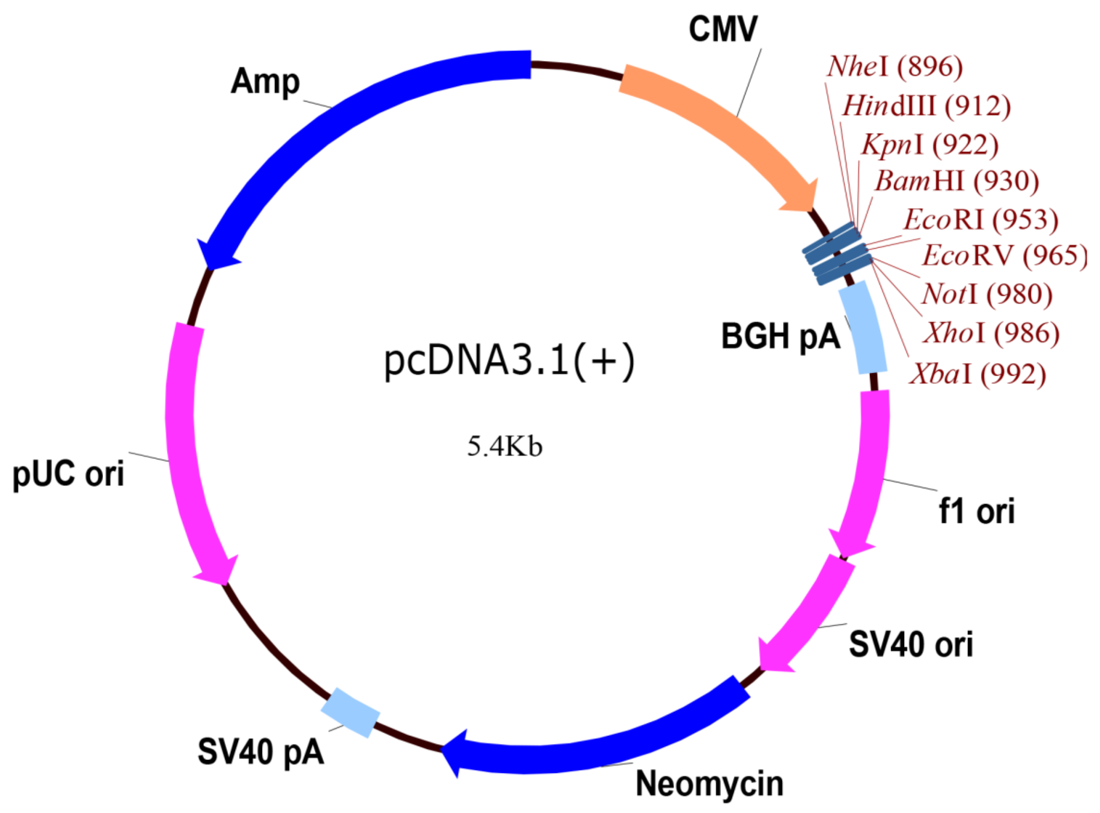

Additional Figure 3: cDNA sequences and plasmid vectors. **a:** ESRP1 3'UTR sequence. **b:** ESRP1 coding region sequence. **c:** hsa_circ_0005585 sequence. **d:** Full design of pmirGLO plasmid vectors. **e:** Full design of ESRP1 overexpresses lentiviral vector. **f:** Full design of hsa_circ_0005585 overexpression plasmid vector.
