# Supplementary figures and images for: High expression of ESRP1 regulated by circ-0005585 promotes cell colonization in ovarian cancer
Source: Cancer Cell Int. 2020 May 19;20:174. doi: 10.1186/s12935-020-01254-3 (PMC7236301; doi:10.1186/s12935-020-01254-3)

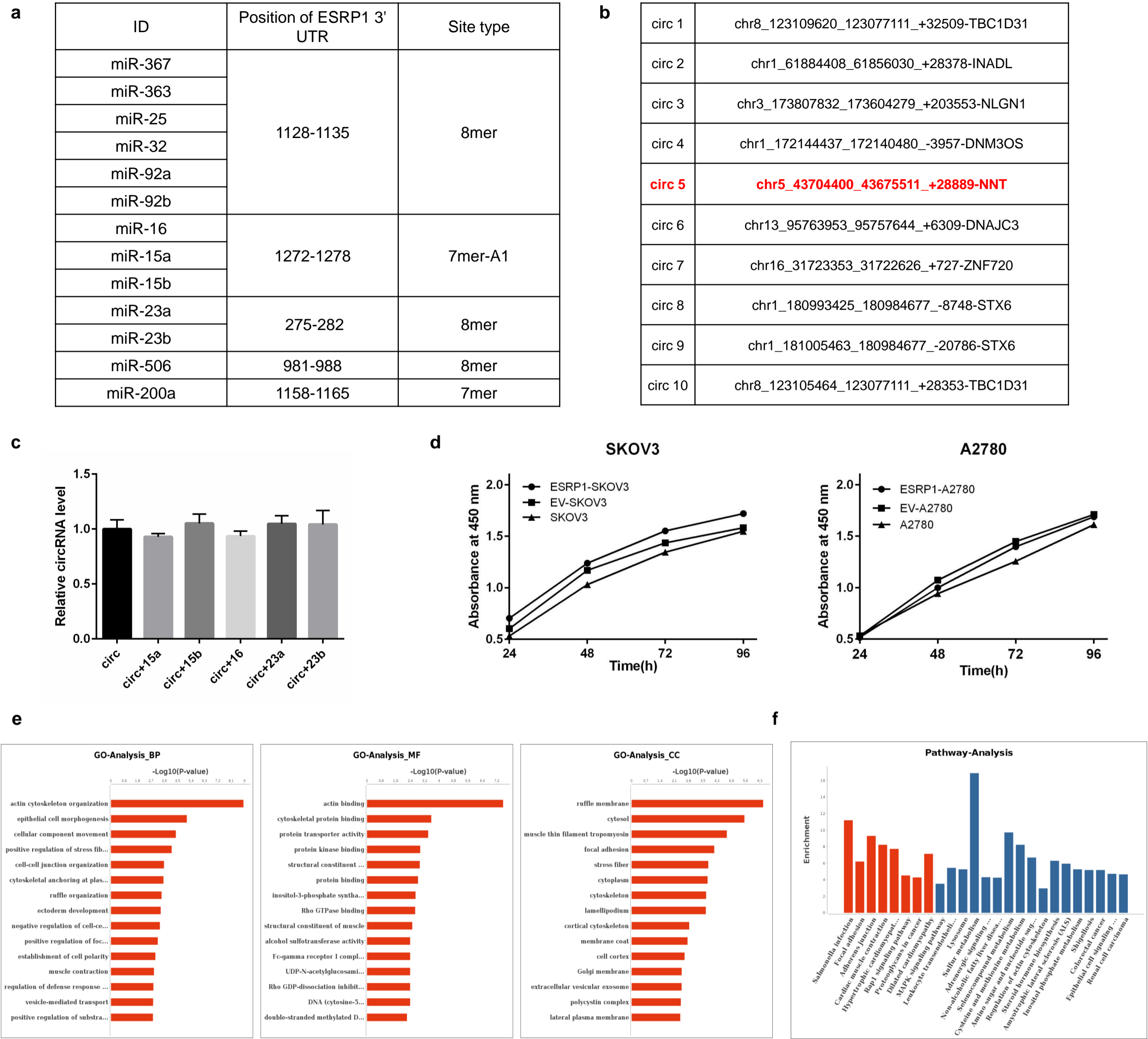

Supplement: Supplementary file 3 — Additional file 3: Figure S2. Non-coding RNA list and ESRP1 bioinformatics functional analysis. [file 12935_2020_1254_MOESM3_ESM.pdf]
