# Supplementary material for: High expression of ESRP1 regulated by circ-0005585 promotes cell colonization in ovarian cancer
Source: Cancer Cell Int. 2020 May 19;20:174. doi: 10.1186/s12935-020-01254-3 (PMC7236301; doi:10.1186/s12935-020-01254-3)
Supplement: Supplementary file 4 — Additional file 4: Figure S3. ESRP1-related pathways. [file 12935_2020_1254_MOESM4_ESM.pdf]

a

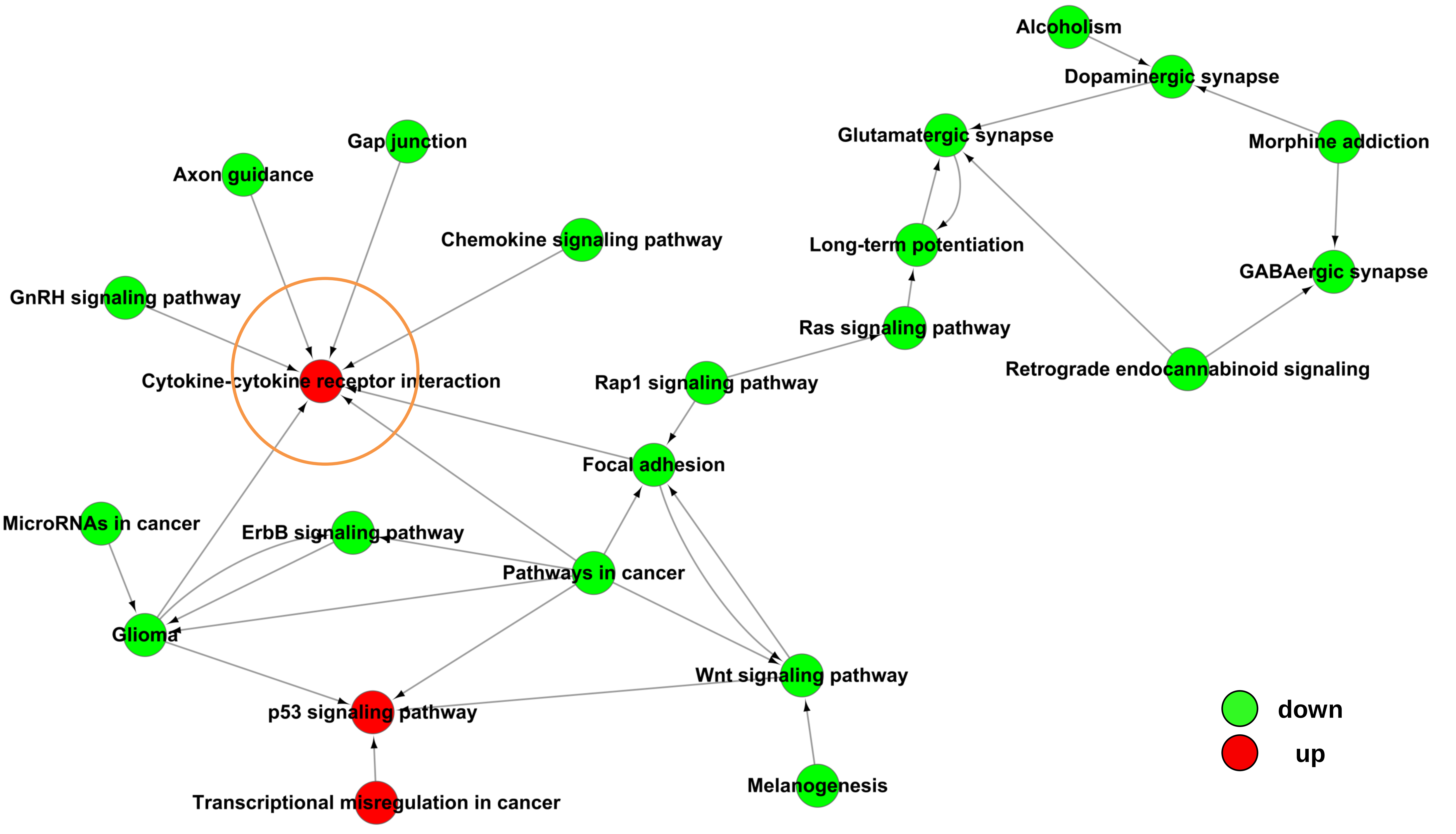

b

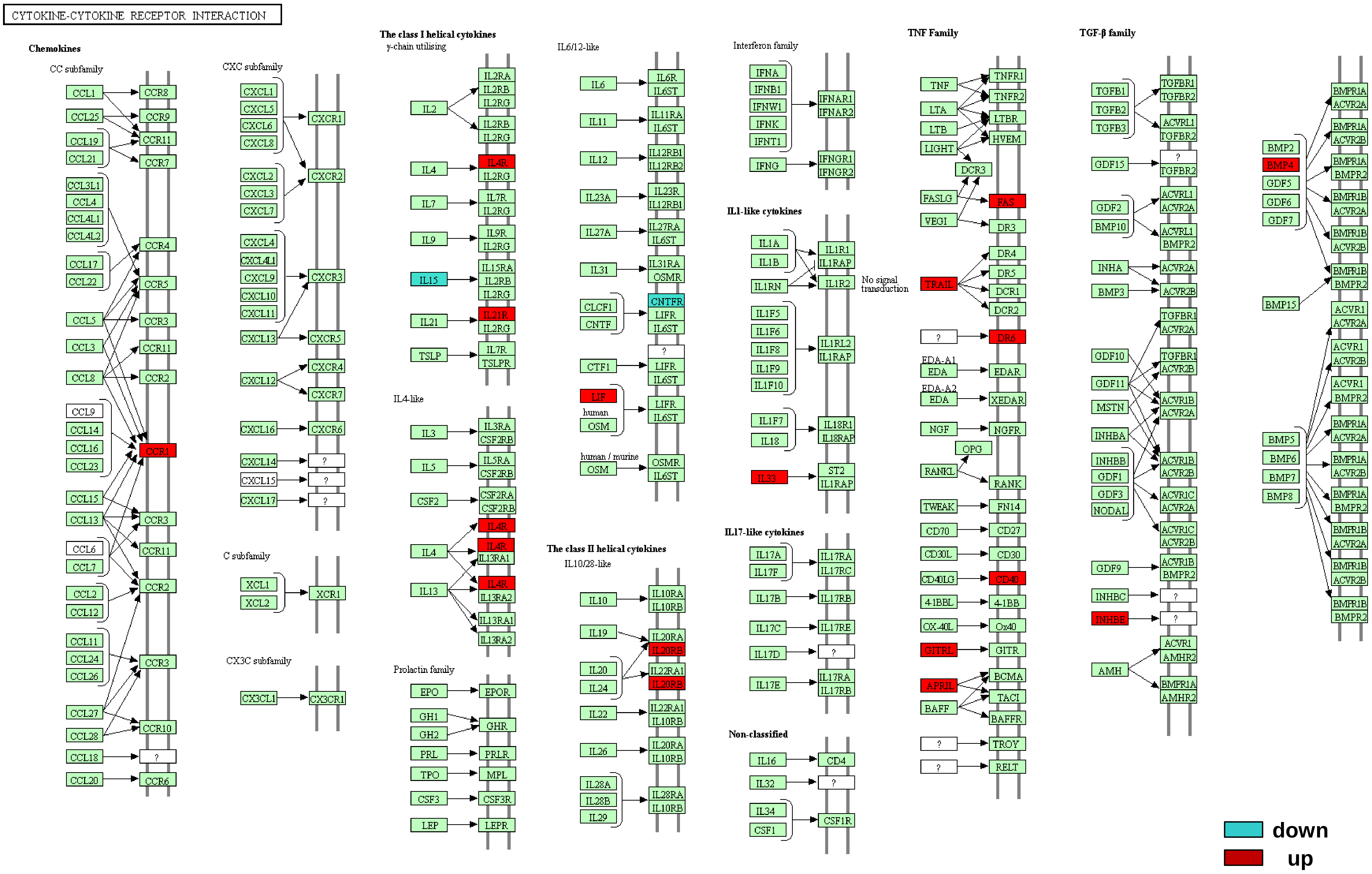

Additional Figure 3: ESRP1-related pathway. **a:** ESRP1 high-throughput sequencing differentially expressed gene-enriched pathways and interrelationships between pathways. **b:** Changes in cytokine-cytokine receptor interaction pathway of ESRP1-SKOV3**.**
